# Extensin and senescence: a cell wall connection

**DOI:** 10.1093/jxb/erad336

**Published:** 2023-09-29

**Authors:** Jasmina Kurepa, Jan A Smalle

**Affiliations:** University of Kentucky, Lexington, KY 40546, USA; University of Kentucky, Lexington, KY 40546, USA

**Keywords:** Arabidopsis, cell wall, extensin, leaf senescence, tomato, ubiquitin ligase

## Abstract

This article comments on:

Lu H, Niu X, Fan Y, Yuan Y, Huang L, Zhao B, Liu Y, Xiao F. 2023. The extensin protein SAE1 plays a role in leaf senescence and is targeted by the ubiquitin ligase SINA4 in tomato. Journal of Experimental Botany 74, 5635–5652.

**Extensins are a family of cell wall proteins that play important roles in plant development and environmental responses. This large protein family is further classified into subfamilies based on the presence or absence of domains in addition to the conserved Ser-(Pro)**_**3–5**_ **motif. The classical extensins stand out among these subfamilies as their functions in plant growth and physiology have remained elusive, predominantly because of perceived genetic redundancy and complications with detecting and assessing their cell wall effects. In this issue, Lu *et al.* (2023) describe a role for the classical extensin SAE1 in tomato leaf senescence. This discovery provides a new avenue for extensin research and uncovers an interesting link between the plant cell wall and leaf senescence timing.**

Extensins are a large superfamily of plant cell wall-localized hydroxyproline-rich glycoproteins that contain a conserved Ser-(Pro)_3–5_ motif (Marzol *et al.,* 2018). The extensin superfamily is subdivided into families whose members share additional constituent domains (Marzol *et al.,* 2018; Castilleux *et al.,* 2021). For example, members of the classical extensin family have a Tyr-containing motif, which is essential for peroxidase-dependent covalent cross-linking of extensin monomers into a complex network (Moussu and Ingram, 2023). The cross-linked extensin network is believed to serve as a scaffold for pectins, hemicellulose, and other cell wall components, and thus is essential for the establishment and maintenance of cell wall structure (Moussu and Ingram, 2023). Although the classical extensins were discovered in 1960, their roles in plant growth and development have remained somewhat elusive, predominantly because of the perceived genetic redundancy of the corresponding gene families (Moussu and Ingram, 2023).

In this issue, Lu *et al.* (2023) show that the classical extensin *SAE1* acts as a promoter of tomato leaf senescence. *SAE1* overexpression accelerates senescence, while *SAE1* loss of function causes a senescence delay. Expression analysis revealed that the *SAE1* gene is down-regulated in mature leaves of wild-type tomato plants, indicating that suppression of SAE1 accumulation is essential for leaf longevity. Moreover, Lu *et al.* (2023) identified a ubiquitin ligase, SISINA4, that suppresses SAE1 accumulation and its senescence-promoting effect. The *SISINA4* gene is up-regulated in mature leaves, thus providing additional control of SAE1 accumulation at later stages of leaf development. These results reveal that SAE1 plays a pivotal role in regulating the initiation of leaf senescence in tomato. Since high-level expression of *SAE1* also caused accelerated senescence in *Arabidopsis thaliana* leaves, the extensin-related senescence mechanism uncovered by Lu *et al.* (2023) may play a critical role in senescence initiation in all dicots.

The cell wall and senescence

Leaf senescence is a complex developmental program essential for managing a plant’s nutrient status and for reallocating resources toward shoot and root growth optimization and maximization of reproductive success (Schippers *et al.,* 2015). Considering the importance and finality of leaf senescence, the discovery that the control of leaf senescence is multilayered and modulated by multiple inputs was not a surprise (Guo *et al.,* 2021). For example, although leaf senescence follows a genetically predisposed timeline, entry into the senescence program can be altered by environmental inputs, which is best illustrated by the accelerated leaf senescence of plants that grow under stress conditions.

Several studies have revealed a link between the cell wall and senescence timing, and identified a number of regulatory proteins as components of senescence signaling pathways (Lee *et al.,* 2020; Li *et al.,* 2021). Although these reports indicated that cell wall characteristics play a role in the timing of senescence, unequivocal proof still awaits cell wall architectural analyses. These studies, however, suggested several hypotheses linking controlled dynamic changes of the cell wall and senescence, which include the enzymatic release of senescence-signaling molecules, changes in cell wall composition, and alterations of cell wall rigidity that trigger a senescence-inducing mechanism.

SAE1 and senescence timing

The *SAE1* gene is down-regulated in mature, pre-senescent leaves at transcriptional and post-translation levels. Together with the finding that plants overexpressing *SAE1* have an accelerated senescence phenotype, this strongly suggests that the accumulation of this classical extensin serves as a senescence trigger and that plants delay their leaf senescence— at least in part—by limiting SAE1 accumulation. As SAE1 is predicted to be a cell wall protein, we can hypothesize that SAE1 influences senescence timing by affecting cell wall structure and, from this perspective, two not mutually exclusive possibilities stand out. First, it is possible that SAE1 directly interacts with a cell membrane-localized senescence signaling mechanism—as pointed out by Lu *et al.* (2023)—and that this mechanism can detect SAE1 levels and induce senescence when SAE1 levels reach a critical threshold (Fig. 1A). A second possibility is that high levels of SAE1 influence the senescence-promoting signaling mechanism indirectly (Fig. 1B). In this case, SAE1-driven changes in cell wall structure are the trigger for the senescence program. Analyses of the role of extensins in pollen tube growth, root growth, and pathogen responses have put forward a hypothesis that cells have detection mechanisms that sense cell wall integrity changes which are based on the interactions of extensin networks with pectins (Marzol *et al.,* 2018). It is conceivable that similar mechanisms also connect cell wall integrity changes with senescence signaling pathways. The link between classical extensins and pectin is particularly interesting from a senescence perspective (Mishler-Elmore *et al.,* 2021). Pectins, which are a major component of land plant cell walls, have multiple regulatory roles in plant development and are known to be targeted by a range of modification enzymes and pectin depolymerases (Shin *et al.,* 2021). Notably, pectin lyases (PELs), a class of pectin depolymerases, have already been implicated in leaf senescence (Leng *et al.,* 2017). Loss of PEL precursor function resulted in increased pectin content combined with accelerated leaf senescence, suggesting that the pectin content of the cell wall plays a role in the timing of senescence (Leng *et al.,* 2017). Future work is expected to reveal the exact mechanisms by which SAE1-dependent changes in cell wall architecture and composition impact senescence timing. Although detecting extensin networks and their interactions with cell wall constituents such as pectin is still experimentally challenging, future work will be greatly facilitated by the genetic resources generated by Lu *et al.* (2023).

**Fig 1.**
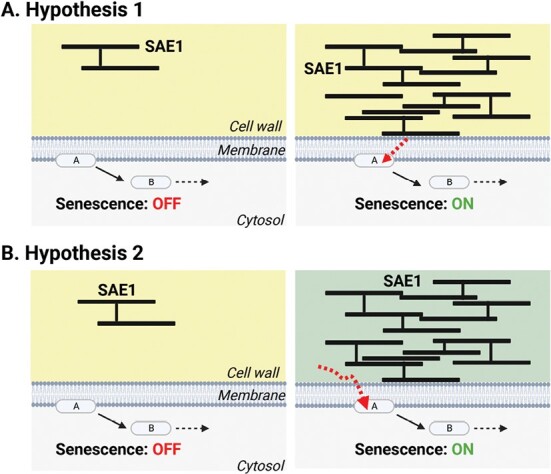
Possible mechanisms underlying SAE1-promoted leaf senescence. (A) Hypothesis 1: cell wall-localized SAE1 interacts with senescence signaling protein A. The senescence program is triggered when the senescence signaling protein is activated beyond a critical threshold. An increase in SAE1 levels therefore is linked with the onset of senescence. (B) Hypothesis 2: increased SAE1 levels lead to an increase in density and complexity of the extensin network, which alters cell wall architecture or composition. This change in cell wall characteristics is detected by senescence signaling protein A and triggers the senescence program.

Another intriguing aspect of this work is that loss of function of a single extensin-encoding gene led to a senescence-delay phenotype, implying an absence of functional redundancy in the tomato classical extensin gene family (Liu *et al.,* 2016). Future work is expected to reveal whether this senescence-delay phenotype is due to differential gene expression (i.e. whether *SAE1* is the most expressed extensin gene during that phase of leaf development) or whether there are indeed functional differences within this gene family. Studies describing the effects of the overexpression of other extensins in Arabidopsis do not report accelerated senescence phenotypes (Roberts and Shirsat, 2006; Wei and Shirsat, 2006), which strengthens the hypothesis that different members of the classical extensin family have distinct functions.

SISINA4 is a RING-type ubiquitin ligase (Wang *et al.,* 2018). Ubiquitin ligases are the components of the ubiquitin–proteasome pathway that provide specificity to this degradation pathway, and their target specificity is narrow (Sharma *et al.,* 2016; Kelley, 2018). Therefore, it will also be of interest to test if SISINA4 specifically targets SAE1 or if it also suppresses the accumulation of other classical extensins. Another open question is whether the SISINA4–SAE1 interaction depends on the secondary modification status of SAE1 and its cellular location, as such interaction requirements would substantially expand the regulatory complexity of this senescence mechanism.

Lastly, the work of Lu *et al.* (2023) has the potential to reveal a module that is specific for leaf development and senescence timing in dicots, as genes encoding classical extensins are largely absent in monocots, gymnosperms, and primitive land plants (Liu *et al.,* 2016). Collectively, this work has opened up various research avenues relevant to extensin and plant senescence biology (Box 1).

**Box 1. Open questions**

Is SAE1 the only senescence-regulating extensin in tomato?

Is SAE1 degradation dependent on secondary modifications and cellular localization?

Is SAE1 a component of a dicot-specific senescence module?

How are SAE1 and SISINA4 integrated in the known leaf senescence regulatory networks?
